# Genetic susceptibility to patient-reported xerostomia among long-term oropharyngeal cancer survivors

**DOI:** 10.1038/s41598-022-10538-9

**Published:** 2022-04-22

**Authors:** Puja Aggarwal, Katherine A. Hutcheson, Robert Yu, Jian Wang, Clifton D. Fuller, Adam S. Garden, Ryan P. Goepfert, Jillian Rigert, Frank E. Mott, Charles Lu, Stephen Y. Lai, G. Brandon Gunn, Mark S. Chambers, Guojun Li, Chih-Chieh Wu, Ehab Y. Hanna, Erich M. Sturgis, Sanjay Shete

**Affiliations:** 1 Department of Epidemiology, The University of Texas MD Anderson Cancer Center, Houston, TX USA; 2 Department of Head and Neck Surgery, The University of Texas MD Anderson Cancer Center, Houston, TX USA; 3 Department of Radiation Oncology, The University of Texas MD Anderson Cancer Center, Houston, TX USA; 4 Department of Biostatistics, The University of Texas MD Anderson Cancer Center, Houston, TX USA; 5 Department of Thoracic Head and Neck Medical Oncology, The University of Texas MD Anderson Cancer Center, Houston, TX USA; 6 Division of Cancer Prevention and Population Sciences, The University of Texas MD Anderson Cancer Center, Houston, TX USA; 7 Department of Environmental and Occupational Health, College of Medicine, National Cheng Kung University, Tainan, 701 Taiwan; 8 Department of Otolaryngology-Head and Neck Surgery, Baylor College of Medicine, Houston, TX USA

**Keywords:** Genetics, Genetic association study, Genome-wide association studies

## Abstract

Genetic susceptibility for xerostomia, a common sequela of radiotherapy and chemoradiotherapy for head and neck cancer, is unknown. Therefore, to identify genetic variants associated with moderate to severe xerostomia, we conducted a GWAS of 359 long-term oropharyngeal cancer (OPC) survivors using 579,956 autosomal SNPs. Patient-reported cancer treatment-related xerostomia was assessed using the MD Anderson Symptom Inventory. Patient response was dichotomized as moderate to severe or none to mild symptoms. In our study, 39.2% of OPC survivors reported moderate to severe xerostomia. Our GWAS identified eight SNPs suggestively associated with higher risk of moderate to severe xerostomia in six genomic regions (2p13.3, rs6546481, Minor Allele (MA) = A, *ANTXR1*, *P* = 4.3 × 10^–7^; 5p13.2–p13.1, rs16903936, MA = G, *EGFLAM*, *P* = 5.1 × 10^–6^; 4q21.1, rs10518156, MA = G, *SHROOM3*, *P* = 7.1 × 10^–6^; 19q13.42, rs11882068, MA = G, *NLRP9*, *P* = 1.7 × 10^–5^; 12q24.33, rs4760542, MA = G, *GLT1D1*, *P* = 1.8 × 10^–5^; and 3q27.3, rs11714564, MA = G, *RTP1*, *P* = 2.9 × 10^–5^. Seven SNPs were associated with lower risk of moderate to severe xerostomia, of which only one mapped to specific genomic region (15q21.3, rs4776140, MA = G, *LOC105370826*, a ncRNA class RNA gene, *P* = 1.5 × 10^–5^). Although our small exploratory study did not reach genome-wide statistical significance, our study provides, for the first time, preliminary evidence of genetic susceptibility to xerostomia. Further studies are needed to elucidate the role of genetic susceptibility to xerostomia.

## Introduction

The incidence of oropharyngeal cancer (OPC) is increasing at a rate of about 5% each year in the United States—faster than any other head and neck cancer (HNC)—and according to current projections, OPC will represent about half of HNCs by 2030^1^. This increased incidence of OPC is attributed to the contribution of the human papillomavirus (HPV) in the etiology of HNC^1–4^. Rates of HPV-associated OPC continue to rise rapidly in a younger, middle-aged population. Fortunately, conventional treatment for HPV-related OPC tumors, often including chemotherapy and/or radiation, is associated with excellent prognosis and decades of potential cancer-free life post-treatment^1–4^. Unfortunately, these patients are at risk of developing substantial cancer treatment-related side-effects, including xerostomia, which often have long-term detrimental impacts on quality of life^1–4^.

Xerostomia is a sensation of dryness in the mouth which, when occurring in HNC cancer patients, is often caused by salivary gland hypofunction secondary to radiotherapy or chemoradiotherapy^5,6^. Radiation-associated xerostomia occurring during or immediately after radiotherapy is commonly referred to as acute xerostomia, which may be caused by salivary gland inflammation^5–7^. In contrast, late xerostomia can occur months after treatment completion and may be caused by fibrosis and damage to the salivary glands, which is often permanent^5–7^. Such radiotherapy-associated salivary gland injury can result in alterations in saliva including volume, pH, and consistency often leading to production of low quantity, high viscosity, and more acidic saliva with drastic impacts on patients’ overall oral health and quality of life^6^.

Despite efforts to prevent radiation-attributable salivary gland injury and resultant xerostomia, patient-reported xerostomia was rated in an earlier study as one of the 5 most prevalent and severely rated cancer treatment-associated symptoms among OPC survivors^8^. In a recent study of OPC patients, 39.1% of OPC survivors reported moderate-to-severe xerostomia which had associations with sex, education level, continued smoking, and bilateral intensity modulated radiotherapy (IMRT) after multivariable adjustment^9^.

Radiation-attributable salivary gland hypofunction and resultant xerostomia often lead to a multifold of downstream negative oral health disorders and dysfunctions including dysphagia (difficulty swallowing), oral pain, voice and speech dysfunction, dysgeusia (change in taste), dental caries, osteoradionecrosis, and oral infections^5–7,10^. These conditions can in turn contribute to dietary alterations, inadequate nutrition secondary to reduced food consumption, loss in body weight, decline in engagement in social activities, increased morbidity, and a decline in quality of life, which is even more devastating given the current lack of effective treatment options for radiation-attributable salivary gland damage and resultant xerostomia^10^. Given lack of treatment options once these conditions develop, efforts need to be aimed at understanding patient risk for radiation-attributable salivary gland damage and xerostomia with high priority on prevention of these conditions.

Genetic susceptibility implicated in development of OPC and oral cancer (OC) were discovered in several loci, including alcohol-related genes (ADH1B and ADH7), and HLA region^11–13^. However, genetic susceptibility to xerostomia is not well understood. Inter-patient heterogeneity/variability to toxicity of normal tissues after radiotherapy contributing to xerostomia in HNC patients has led to postulations of potential genetic influence on the radiosensitivity of irradiated tissues^14–16^. Genes involved in inflammatory pathways, tumorigenesis, and detection and repair of DNA damage may lead to functional changes/consequences in downstream protein, potentially impairing the ability to repair DNA damage^14–16^. This could contribute to alteration of intrinsic radiosensitivity of irradiated tissues and subsequent radiotherapy-associated adverse effects including xerostomia^17^. It has been reported that DNA sequence variation/polymorphism in genes, including *XRCC3* and *ATM* (responsible for DNA repair) and *TGFβ1* (linked with fibrinogenesis/proliferation of fibroblasts), are correlated with radiotherapy-related hypersensitivity^18–20^.

To our knowledge no study has investigated genetic basis of xerostomia using a genome-wide study. The objective of this study was to identify genetic predictors of susceptibility to patient-reported cancer treatment-associated xerostomia among long-term OPC survivors using genome-wide association methods. We hypothesized that genetic variants are associated with cancer treatment-associated xerostomia. Identification of genetic variants associated with xerostomia would allow risk stratification as well as targeted efforts aimed at prevention, surveillance, and management.

## Material and methods

### Study population

**Figure 1:**
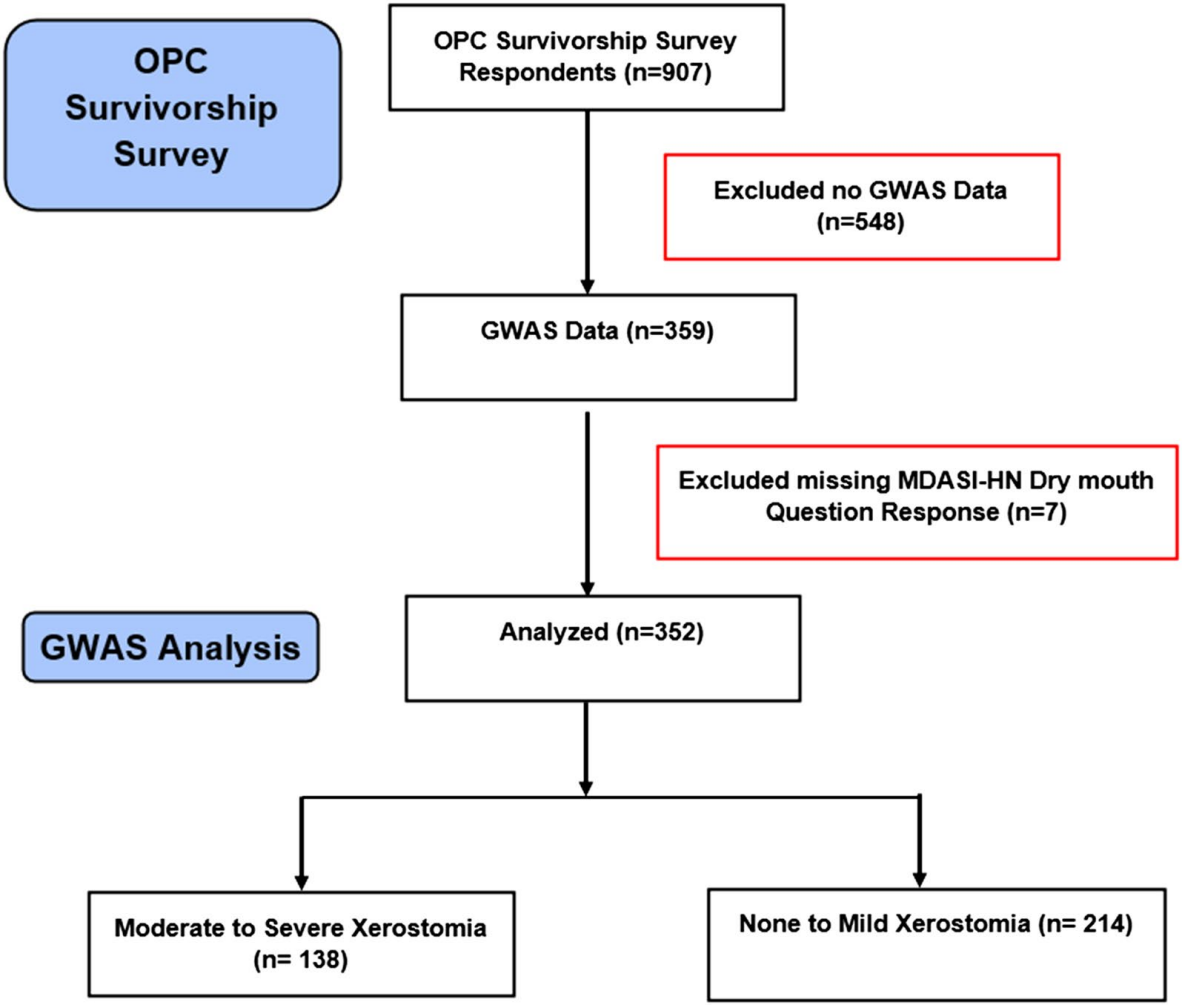
Consort figure.

This study population included OPC survivors treated from January 1, 2000, to December 31, 2013, at The University of Texas MD Anderson Cancer Center. Study participants were 18 years or older and responded to an OPC survivorship survey at least 1 year after curative OPC treatment completion. Exclusion criteria included the following: recurrent HNC, second primary tumors, and distant metastasis. Figure 1 shows the ascertainment/recruitment of the study population. Written informed consent was obtained from all study participants for collection of blood samples and clinic-demographic information and a consent statement was used on survey cover letter as written survey informed consent. The study protocols PA11-0936 and LAB00-062 were approved by the Institutional Review Board at The University of Texas MD Anderson Cancer Center in accordance with tenets of the Declaration of Helsinki.

### Primary outcome

Patient-reported cancer treatment-related xerostomia was the primary outcome variable in this study. The outcome variable was defined using the MD Anderson Symptom Inventory Head and Neck Cancer Module (MDASI-HN)^21^, a validated multi-symptom instrument that asked respondents: “How severe are your symptoms? People with cancer frequently have symptoms that are caused by their disease or their treatment^21^. We ask you to rate how severe the symptoms have been in the last 24 h.” Subsequently, respondents were asked to score the intensity of xerostomia experienced with “having dry mouth at its worst”. Response categories ranged from 0 (“not present”) to 10 (“as bad as you can imagine”)^21^. Patient response to the dry mouth question was then dichotomized to following categories: 0–4 were coded as none to mild, and 5–10 were coded as moderate to severe^22,23^. We dichotomized the xerostomia into clinically meaningful categories: moderate to severe versus none to mild.

### Genotyping

The genotype data for OPC patients was obtained from our previous study which included patients with newly or recently diagnosed and previously untreated squamous cell carcinoma of the head and neck^24^, ascertained at Head and Neck Surgery Clinic at The University of Texas MD Anderson Cancer Center, Texas^25,26^. The Illumina HumanOmniExpress-12v1 BeadChip was used for genotyping of the genomic DNA. Using the same quality control of the genotype data as previously described (inclusive of use of genotype data to identify participants with discordant sex classification, those who were genetically related, and those that were duplicates)^24^, we had genotype data on a total 579,956 autosomal SNPs.

### Clinical and sociodemographic covariates

Information on demographic variables including age at OPC diagnosis, sex, and education level; clinical variables including primary tumor subsite, T and N staging, survival time, HPV status, cigarette smoking status at time of cancer diagnosis, and ability to eat a solid food diet prior to treatment; and treatment-related variables including treatment modality, receipt of chemotherapy, surgery, neck dissection, and radiotherapy dose, fractionation schedule, and type were abstracted from patients’ electronic medical charts. Primary head and neck tumor subsites included tonsil, base of tongue and glossopharyngeal sulcus, and others (including soft palate, pharyngeal wall, and oropharynx site not otherwise specified). T and N staging was according to the American Joint Committee on Cancer 7th edition criteria. Survival time was calculated as the difference between age at time of diagnosis and age at time of survey. Radiotherapy information included the following: total radiation dose to primary tumor measured in Gray (Gy); radiotherapy fractionation schedule including standard fractionation (70.0 Gy given in 33–35 fractions), accelerated fractionation (72.0 Gy given in 40 fractions or use of concomitant boost or Danish Head and Neck Cancer Group regimens), and no radiotherapy; and radiotherapy type. Radiotherapy types were categorized as three-dimensional conformal radiotherapy (3D-CRT), bilateral IMRT with split-field or whole-field IMRT, volumetric-modulated arc therapy, and proton therapy; and ipsilateral IMRT regimens. Also, as parotid glands produce 60–65% of saliva^27^ and mean dose to parotid glands of ≤ 26 Gy can contribute to preserving salivary flow^28^. Therefore, we also abstracted information on mean dose to parotid glands from medical charts and, using the threshold dose, categorized mean parotid gland dose as follows; ≤ 26 Gy, > 26 Gy, and missing/don’t know. Cigarette smoking status was determined as follows: participants who had not smoked 100 cigarettes in their lifetime were classified as never smokers, those who had quit more than 6 months before diagnosis were considered former smokers at the time of diagnosis^20,29,30^ and finally, current smokers at the time of diagnosis were further categorized into those who quit subsequently and those who continued to smoke^9,23^.

### Statistical methods

Descriptive statistics were used to summarize the study data and the Kruskal Wallis test and Fishers exact test were used to test for differences between xerostomia categories for continuous and categorical variables, respectively. In a larger study, we had found that sex, education, cigarette smoking, and radiotherapy type were significantly associated with xerostomia; therefore, we adjusted our genetic analyses with these covariates^9^ along with mean dose to parotid glands^28^. Quality control for genotype data in our study included: removing SNPs with Hardy–Weinberg equilibrium (HWE; *P* < 1 × 10^–6^), genotyping typing call rate ≤ 95%, and minor allele frequency ≤ 0.05 among OPC patients^24^. The pairwise genetic distance among the 359 OPC patients was estimated using identity-by-state (IBS) methods implemented in PLINK (v1.90b3.34 64-bit [15 Mar 2016])^26^. A clustering variable was created to identify genetically related/similar individuals to take into consideration underlying population substructure^24^. This clustering approach is a standard established approach for population stratification^31^. In particular, the complete linkage agglomerative clustering is used, based on pairwise IBS distance, with some restrictions, including no merged clusters that contain significantly different patients based on a pairwise population concordance test (*P* < 0.001), all pairs of patients containing at least one case and one control, and cluster size restrictions. The clustering variable created in this way represents the fine-scale population structure of the ancestry and was incorporated in the analysis as a covariate to adjust for the potential underlying population substructure.

Exact logistic regression analysis was conducted using an additive genetic model adjusting for covariates of sex, two-level education variable, three-level radiotherapy type variable, four-level cigarette smoking variable, and the genetic cluster variable described earlier. Odds ratios (ORs) and their corresponding 95% confidence intervals (CIs) were estimated. As a preliminary exploratory study with a small sample, our goals remained hypothesis generating and were focused on identification of genetic variants with potential associations with moderate to severe xerostomia. Accordingly, *P* < 1 × 10^–5^ was used as a criterion for selecting and reporting SNPs, as adjustment for multiple comparisons testing was not feasible. PLINK (v1.9/2.0, https://www.cog-genomics.org/plink/2.0/ v1.90b3.34 64-bit. https://www.cog-genomics.org/plink2 ) software was used for association analysis^31^, and Manhattan plots were constructed in R with custom function and the calibrate package. To further understand potential role and function of identified SNPs at mapped locations in relation to genes, function, and downstream effects, GeneCards (https://www.genecards.org/), dbSNP (https://www.ncbi.nlm.nih.gov/snp/), NCBI Aceview (https://www.ncbi.nlm.nih.gov/IEB/Research/Acembly/), and PubMed (https://pubmed.ncbi.nlm.nih.gov/) were accessed and comprehensively searched. We also conducted the gene set enrichment analysis (GSEA) using the extension to gene set enrichment analysis approach, GSEA-SNP^32,33^.

Additionally, we explored the cumulative contribution of SNPs to moderate to severe xerostomia. Specifically, we calculated polygenic score (PGS), which accounts for multiple statistically significant SNPs and assessed the association between PGS and risk of moderate to severe xerostomia. For *k* independent significant SNPs associated with risk of moderate to severe xerostomia, the PGS was calculated as $PGS={\sum }_{i=1}^{k}{\beta }_{i}{N}_{i}$, where ${\beta }_{i}$ is the coefficient estimate of SNP *i* (i.e., log(OR_*i*_)) from a logistic regression analysis and ${N}_{i}$ is the number of minor alleles for SNP *i*. Two PGSs were created to take into consideration protective SNPs (i.e., OR < 1; less likely to develop moderate to severe xerostomia) and risk SNPs (i.e., OR ≥ 1; more likely to develop moderate to severe xerostomia), respectively. Finally, we estimated the heritability for xerostomia using the software tool genome-wide complex trait analysis (GCTA)^34,35^, with adjustment for the covariates (i.e., sex, education, RT type, mean parotid gland dose, population substructure cluster).

## Results

### Characteristics of the study populations

Table 1 summarizes the distribution of demographic, clinical, and treatment-related characteristics of this study population. Our study population included 359 patients with OPC, seven of whom did not respond to the MDASI-HN xerostomia question. Majority of OPC survivors in our study were male (85.8% = 308/359). Among the 352 patients who responded to the xerostomia question, 138 (39.2%) reported moderate to severe xerostomia and 214 (60.8%) reported none to mild xerostomia. There were no significant differences by age among xerostomia sub-groups. Higher proportions of female patients (27; 52.9%) and those with less than high school education (32; 54.2%) reported moderate to severe xerostomia, and these differences were statistically significant. Interestingly, most patients with moderate to severe xerostomia had T1 and T2 tumors (96; 69.6%), were never and former smokers (114; 82.6%), received standard radiotherapy fractionation (113; 81.9%), and were treated with IMRT (121; 87.7%) regimens. Median radiotherapy dose was significantly higher among patients with moderate to severe xerostomia than in patients with none to mild xerostomia (70.0 Gy vs. 66.0 Gy), and a significantly higher proportion of those who received 3D-CRT (17/28; 63.0%) reported moderate to severe xerostomia. Mean parotid gland dose was available for 243 patients and missing for 116 of them. Of these 243 patients, mean parotid gland dose was > 26 Gy for 96 (39.5%) patients of which 42 (43.8%) reported moderate to severe xerostomia. Among the patients with moderate to severe xerostomia, significantly higher proportion of those who received multimodality chemotherapy treatment (103; 43.1%) reported moderate to severe xerostomia in comparison to those treated with single modality regimens. The pairwise-IBS-distance-based clustering analysis resulted in 13 clusters. The frequencies and percentages of the clusters underlying population substructure were similar in patients with none to mild xerostomia and patients with moderate to severe xerostomia. This clustering variable was adjusted for in the analysis as a covariate. Lastly, using GCTA, we found the heritability of xerostomia to be 26%, given the prevalence of moderate to severe xerostomia of 39% observed in our data.

**Table 1:** Characteristics and distribution of oropharyngeal cancer patients by clinical and demographic factors.

Variables	All patients (N = 359)	Xerostomia information missing (N = 7)	None to mild xerostomia (N = 214)	Moderate to severe xerostomia (N = 138)
**Age at diagnosis**, y, median (range, IQR)			55 (34–80, 50–60)	55 (34–73, 50–61)
**Survival time**, y, median (range, IQR)			8 (2–16, 6–11)	8 (3–15, 5–11)
**Radiation dose**, Gy, median (range, IQR)			66 (60–72, 66–70)	70 (40–72.6, 66–70)
**Sex, No. (%)**				
Female	51 (14.2)	0	24 (47.1)	27 (52.9)
Male	308 (85.8)	7	190 (63.1)	111 (36.9)
**Education, No. (%)**				
> Highschool	272 (75.8)	6	170 (63.9)	96 (36.1)
≤ Highschool	59 (16.4)	0	27 (45.8)	32 (54.2)
Missing	28 (7.8)	1	17 (63.0)	10 (37.0)
**Race/ethnicity, No. (%)**				
Non-Hispanic white	348 (97.0)	7	209 (61.3)	132 (38.7)
Non-Hispanic black	1 (0.3)	0	0 (0.0)	1 (100.0)
Hispanic	3 (0.8)	0	2 (66.7)	1 (33.3)
Missing	7 (1.9)	0	3 (42.9)	4 (57.1)
**Primary site, No. (%)**				
Tonsil	157 (43.7)	2	96 (61.9)	59 (38.1)
Base of tongue, GPS	189 (52.7)	5	110 (59.8)	74 (40.2)
Other	13 (3.6)	0	8 (61.5)	5 (38.5)
**T classification, No. (%)**				
1	125 (34.8)	3	79 (64.8)	43 (35.2)
2	136 (37.9)	3	80 (60.2)	53 (39.8)
3	61 (17.0)	0	34 (55.7)	27 (44.3)
4	37 (10.3)	1	21 (58.3)	15 (41.7)
**N classification, No. (%)**				
N0	38 (10.6)	2	21 (58.3)	15 (41.7)
N1 + 2a	92 (25.6)	3	62 (69.7)	27 (30.3)
2b + 3	175 (48.8)	2	100 (57.8)	73 (42.2)
2c	54 (15.0)	0	31 (57.4)	23 (42.6)
**HPV status, No. (%)**				
Negative	26 (7.2)	0	15 (57.7)	11 (42.3)
Positive	124 (34.5)	1	75 (61.0)	48 (39.0)
Unknown	209 (58.2)	6	124 (61.1)	79 (38.9)
**Cigarette smoking, No. (%)**				
Never	162 (45.1)	2	101 (63.1)	59 (36.9)
Former smoker at time of diagnosis	149 (41.5)	5	89 (61.8)	55 (38.2)
Quit smoking subsequent to diagnosis	35 (9.8)	0	17 (48.6)	18 (51.4)
Current smoker at time of survey	9 (2.5)	0	4 (44.4)	5 (55.6)
Don’t know	4 (1.1)	0	3 (75.0)	1 (25.0)
**Solid food pretreatment, No. (%)**				
Yes	356 (99.2)	6	212 (60.6)	138 (39.4)
No	3 (0.8)	1	2 (100.0)	0 (0.0)
**Treatment group, No. (%)**				
Single modality	118 (32.9)	5	78 (69.0)	35 (31.0)
Multimodality	241 (67.1)	2	136 (56.9)	103 (43.1)
**Chemotherapy, No. (%)**				
No	118 (32.9)	5	78 (69.0)	35 (31.0)
Yes	241 (67.1)	2	136 (56.9)	103 (43.1)
**Surgery, No. (%)**				
No	357 (99.4)	7	213 (60.9)	137 (39.1)
Yes—robotic	1 (0.3)	0	1 (100.0)	0 (0.00)
Yes—open	1 (0.3)	0	0 (0.00)	1 (100.0)
**Neck dissection, No. (%)**				
No	281 (78.3)	7	166 (60.6)	108 (39.4)
Yes	78 (21.7)	0	48 (61.5)	30 (38.5)
**RT schedule, No. (%)**				
Standard fractionation	302 (84.1)	6	183 (61.8)	113 (38.2)
Accelerated	57 (15.9)	1	31 (55.4)	25 (44.6)
Missing/no RT	0 (0.0)		0 (0.0)	0 (0.0)
**RT type, No. (%)**				
3D-CRT	28 (7.8)	1	10 (37.0)	17 (63.0)
IMRT Bilateral (SF + IMRT + WF + VMAT) + Proton	299 (83.3)	4	183 (62.0)	112 (38.0)
IMRT ipsilateral	32 (8.9)	2	21 (70.0)	9 (30.0)
Missing/no	0 (0.0)		0 (0.0)	0 (0.0)
**Parotid gland dose, No. (%)**				
Mean parotid gland dose ≤ 26 Gy	147 (41.0)	3	93 (64.6)	51 (35.4)
Mean parotid gland dose > 26 Gy	96 (26.7)	0	54 (56.2)	42 (43.8)
Missing/Don't Know	116 (32.3)	4	67 (59.8)	45 (40.2)
**Cluster for population stratification, No. (%)**				
Cluster 1	69 (19.2)	2	39 (18.2)	28 (20.3)
Cluster 2	45 (12.5)	0	26 (12.1)	19 (13.8)
Cluster 3	34 (9.5)	0	24 (11.2)	10 (7.2)
Cluster 4	33 (9.2)	1	20 (9.3)	12 (8.7)
Cluster 5	37 (10.3)	0	24 (11.2)	13 (9.4)
Cluster 6	29 (8.1)	0	16 (7.5)	13 (9.4)
Cluster 7	8 (2.2)	0	4 (1.9)	4 (2.9)
Cluster 8	28 (7.8)	1	15 (7.0)	12 (8.7)
Cluster 9	50 (13.9)	2	33 (15.4)	15 (10.9)
Cluster 10	16 (4.5)	0	10 (4.7)	6 (4.3)
Cluster 11	8 (2.2)	1	3 (1.4)	4 (2.9)
Cluster 12	1 (0.3)	0	0 (0.0)	1 (0.7)
Cluster 13	1 (0.3)	0	0 (0.0)	1 (0.7)

3D-CRT, three-dimensional conformal radiotherapy; GPS, glossopharyngeal sulcus; IMRT-SF, intensity-modulated radiotherapy split-field technique; IMRT-WF, intensity-modulated radiotherapy whole-field technique; IQR, interquartile range; RT, radiotherapy; VMAT, volumetric-modulated arc therapy.

### Association analysis

Table 2 lists the top 15 SNPs associated with moderate to severe xerostomia among OPC survivors in our study (*P* < 1 × 10^–5^). Among them eight SNPs were associated with increasing odds of reporting moderate to severe xerostomia; of which two did not match to any specific gene region. The remaining seven SNPs were associated with lower odds of reporting moderate to severe xerostomia; of which, six SNPs did not match to any specific gene region.

**Table 2:** Association results of leading SNPs with *P* ≤ 1 × 10^−5^ associated with moderate to severe xerostomia among oropharyngeal cancer survivors.

SNP	Chr	Base-pair position	Minor allele	Odds ratio	L95	U95	*P*	Frequency of minor allele in moderate to severe xerostomia	Frequency of minor allele in none to mild xerostomia	Frequency of minor allele in 1000 genomes project	Gene	Gene location	Function or disease associated
rs6546481	2	69,313,511	A	4.70	2.50	8.83	4.3 × 10^–7^	0.05	0.06	0.11	*ANTXR1* (anthrax toxin receptor)	2p13.3	Role in cellular invasion and metastasis in NPC, colorectal cancer, gastric cancer, oropharyngeal anthrax, gingival disorders
rs16903936	5	38,322,975	G	3.98	2.16	7.31	5.1 × 10^–6^	0.20	0.06	0.14	*EGFLAM (EGF Like, Fibronectin Type III and Laminin G Domains):* intron variant	5p13.2–p13.1	Role in matrix assembly and cell adhesiveness. Involved in ovarian cancer, non-Hodgkin lymphoma and expression correlates with cell proliferation, migration, invasion and poor prognosis in Glioblastoma
rs10518156	4	77,695,104	G	6.65	2.65	16.69	7.1 × 10^–6^	0.13	0.02	0.06	*SHROOM3* (shroom family member 3)	4q21.1	Role in regulation of cell shape changes and binding of actin, proteins, beta-catenins, and actin filament and ligand-gated sodium channel activity. Acute myeloid leukemia, neutrophil actin dysfunction, neural tube defects, atrial septal defect 2. *SHROOM2* (paralog to SHROOM3): prostate carcinoma, colorectal cancer
rs746154	15	70,677,754	A	0.31	0.18	0.52	8.0 × 10^–6^	0.31	0.59	0.42	NA	NA	NA
rs1038553	15	53,679,121	G	0.27	0.14	0.50	9.5 × 10^–6^	0.07	0.22	0.21	NA	NA	NA
rs4776140	15	53,680,596	G	0.27	0.15	0.51	1.5 × 10^–5^	0.07	0.22	0.21	*LOC105370826:* 2 KB upstream variant	15q21.3	LOC105370826 (Uncharacterized LOC105370826) is an RNA Gene and is affiliated with the ncRNA class
rs11882068	19	56,227,165	G	3.53	1.96	6.37	1.7 × 10^–5^	0.20	0.07	0.10	*NLRP9 (*NLR family pyrin domain containing 9)	19q13.42	Role in the innate immune system regulation and inflammation
rs4760542	12	129,385,824	G	2.94	1.80	4.80	1.8 × 10^–5^	0.27	0.11	0.18	*GLT1D1* (glycosyltransferase 1 domain containing 1)	12q24.33	Oncogene for colorectal cancer, hepatocellular carcinoma. GenomeRNAi human phenotypes included diminished HPV16 pseudovirus infection
rs3014269	1	227,539,205	G	4.08	2.11	7.89	2.1 × 10^–5^	0.18	0.05	0.08	NA	NA	
rs7523492	1	157,637,964	G	0.42	0.28	0.64	2.5 × 10^–5^	0.26	0.46	0.38	NA	NA	NA
rs1486548	15	53,665,247	A	0.30	0.17	0.54	2.7 × 10^–5^	0.08	0.23	0.21	NA	NA	
rs11714564	3	186,918,213	G	3.05	1.80	5.17	2.9 × 10^–5^	0.24	0.09	0.17	*RTP1*: Receptor Transporter Protein 1,3 Prime UTR Variant *LOC101929106*: Intron Variant	3q27.3	Role in olfactory receptor binding and may be involved with bitter taste receptors. Olfactory receptors may play a role in tumorigenesis have been implicated in melanoma, invasive breast cancer, endometrial cancer, and have also been implicated in prostate cancer cell proliferation where they may be a potential biomarker for patient outcomes
rs10219117	10	92,022,480	A	0.39	0.24	0.61	3.1 × 10^–5^	0.17	0.35	0.28	NA	NA	NA
rs12565883	1	20,883,203	G	0.38	0.24	0.61	3.3 × 10^–5^	0.15	0.33	0.29	NA	NA	NA
rs1158267	11	44,396,537	A	5.06	2.30	11.12	3.5 × 10^–5^	0.24	0.06	0.12	NA	NA	NA
PGS-protective	–	–	–	0.58	0.48	0.70	1.6 × 10^–8^	–	–	–	NA	NA	NA
PGS-risk	–	–	–	2.59	1.87	3.59	3.4 × 10^–8^	–	–	–	NA	NA	NA

Abbreviations: NPC, nasopharyngeal cancer; PGS, polygenic score.

The leading top-ranked SNP in our study (rs6546481, OR 4.70, 95% CI 2.50–8.83, *P* = 4.3 × 10^–7^) was located on chromosome 2 and was mapped to the *ANTXR1* (anthrax toxin receptor 1) gene. *ANTXR1* has been previously associated with metastasis in head and neck cancer patients with nasopharyngeal tumors and other oral disorders^38,39^.

OPC survivors with at least one copy of minor allele G for rs16903936 were associated with higher odds of reporting moderate to severe xerostomia (OR 3.98, 95% CI 2.16–7.31, *P* = 5.1 × 10^–6^). This SNP was mapped to the *EGFLAM* (EGF Like, Fibronectin Type III and Laminin G Domains) gene which has been associated with ovarian cancer, non-Hodgkin lymphoma, and Glioblastoma^40^.

Carriers of least one copy of minor allele G for rs10518156 were associated with higher odds of reporting moderate to severe xerostomia (OR 6.65, 95% CI 2.65–16.69, *P* = 7.1 × 10^–6^). This SNP was mapped to the *SHROOM3* (shroom family member 3) gene, which has been associated with acute myeloid leukemia, neural tube defects, and atrial septal defects^41^.

Study participants with at least one copy of minor allele G for rs11882068 were associated with higher odds of reporting moderate to severe xerostomia (OR 3.53, 95% CI 1.96–6.37, *P* = 1.7 × 10^–5^). This SNP was mapped to the *NLRP9* (NLR family pyrin domain containing 9) gene, which may play an important role in the innate immune system regulation and inflammation^42^.

Carriers of at least one copy of minor allele G for rs4760542 were associated with higher odds of reporting moderate to severe xerostomia (OR 2.94, 95% CI 1.80–4.80, *P* = 1.8 × 10^–5^). This SNP was mapped to the *GLT1D1* (glycosyltransferase 1 domain containing 1) gene, which is postulated to be an oncogene for colorectal cancer and associated with hepatocellular carcinoma^43^.

Study participants with at least one copy of minor allele G for rs11714564 were associated with higher odds of reporting moderate to severe xerostomia (OR 3.05, 95% CI 1.80–5.17, *P* = 2.9 × 10^–5^). This SNP was mapped to the *RTP1* (Receptor Transporter Protein 1) gene, which plays a role in binding of olfactory receptor genes which are involved in several cancers^44^.

Also, rs3014269 (OR 4.08, 95% CI 2.11–7.89, *P* = 2.1 × 10^–5^), and rs1158267 (OR 5.06, 95% CI 2.30–11.12, *P* = 3.5 × 10^–5^) were associated with increased odds but were not mapped to any specific gene region.

Among SNPs associated with lower odds of moderate to severe xerostomia, only one rs4776140 (OR 0.27, 95% CI 0.15–0.51, *P* = 1.5 × 10^–5^) was mapped to LOC105370826, a ncRNA class RNA gene^45^. Additionally, r rs746154 (OR 0.31, 95% CI 0.18–0.52, *P* = 8.0 × 10^–6^), rs1038553 (OR 0.27, 95% CI 0.14–0.50, *P* = 9.5 × 10^–6^), rs7523492 (OR 0.42, 95% CI 0.28–0.64, *P* = 2.5 × 10^–5^), rs1486548 (OR 0.30, 95% CI 0.17–0.54, *P* = 2.7 × 10^–5^), rs10219117 (OR 0.39, 95% CI 0.24–0.61, *P* = 3.1 × 10^–5^), and rs12565883 (OR 0.38, 95% CI 0.24–0.61, *P* = 3.3 × 10^–5^) were all associated with lower odds but were not mapped to any specific gene region.

Furthermore, the analysis results using PGS to explore the top-ranked SNPs in a combinatorial manner were reported in Table 2. The PGS was calculated respectively for eight risk-associated SNPs and seven protectively-associated SNPs. We observed statistically significant associations between PGSs and likelihood of developing moderate to severe xerostomia. The PGS combining eight risk SNPs was associated with moderate to severe xerostomia with *P* = 3.4 × 10^–8^. Similarly, the PGS combining seven protective SNPs was also significantly associated with moderate to severe xerostomia with *P* = 1.6 × 10^–8^.

**Figure 2:**
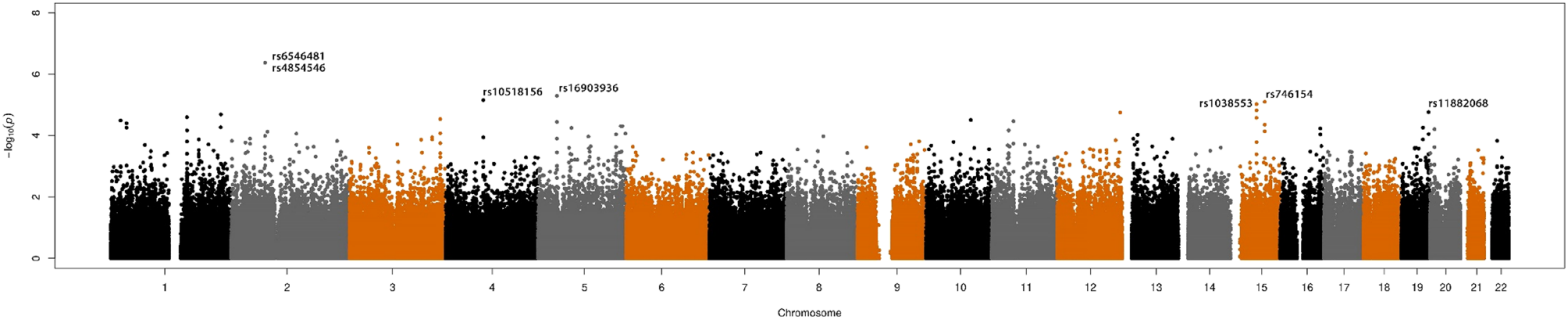
Manhattan plots of the genome-wide association analysis with moderate to severe xerostomia among oropharyngeal cancer survivors. The *y*-axis represents the − log_10_ *P* values.

Supplementary Information Table S1 lists the top 100 SNPs (*P* < 1 × 10^–4^) associated with moderate to severe xerostomia among OPC survivors. Figure 2 shows the Manhattan plots of the GWAS association analysis with moderate to severe xerostomia in our study. We also conducted the GSEA analysis using the six genes identified based on the top-ranked SNPs associated with moderate to severe xerostomia (Table 2). In our GWAS data, a total of 295 SNPs mapping to these six genes were used to conduct pathway-driven analysis using an extension to the gene set enrichment analyses approach, GSEA-SNP. We used Molecular Signatures Database in combination with HP_XEROSTOMIA database^36,37^. The results for top 25 gene sets using the GSEA-SNP analysis are listed in the Supplementary Information Table S2. No significant enrichment for any gene set was observed.

As an additional assessment of role of genetics to the xerostomia phenotype, we also conducted GWAS analyses with two re-categorized xerostomia phenotypes. In particular, we first dichotomized the xerostomia as mild to severe (1–10) versus none (0), and then as moderate to severe (5–10) versus none (0). Sex, education, RT type, mean parotid gland dose and cluster variable for population substructure were adjusted for in both analyses. The top 50 SNPs associated with the two re-categorized xerostomia phenotypes are listed in Supplementary Information Tables S3 and S4, respectively.

Lastly, we also assessed the association between the 15 top-ranked SNPs and dysphagia, a symptom of difficulty in swallowing which is related to xerostomia. The patient response to dysphagia was dichotomized to two categories: none to mild (0–4) and moderate to severe (5–10). Sex, education, RT type and cluster variable for population substructure were adjusted for in the analysis. The results are reported in Supplementary Information Table S5. The top significant SNP associated with dysphagia was rs1158267 (OR 3.31, 95% CI 1.70–6.46, *P* = 9.4 × 10^–4^).

## Discussion

Xerostomia is a common complication/sequela of HNC treatment often resulting in functional impairment and debilitating morbidity. To the best of our knowledge, this is the first study to conduct a genome-wide association analysis to identify genetic variants associated with risk of moderate to severe patient-reported xerostomia among OPC survivors. In this small exploratory GWAS study, we identified 15 SNPs with potential associations with moderate to severe xerostomia; seven of the SNPs belong to specific genomic regions (2p13.3, 3q27.3, 4q21.1, 5p13.2–p13.1, 12q24.33, 15q21.3, 19q13.42). Of the 15 variants, 8 were associated with higher risk, and 7 were associated with lowering risk of moderate to severe xerostomia. The most prominent findings in our study included potential associations of *ANTXR1, RTP1*, *GLT1D1*, *NLRP9,* and *EGFLAM* genes with xerostomia. Although our small sample study did not reach the genome-wide statistical significance (5.0 × 10^–8^), our study provides preliminary evidence of genetic basis for xerostomia which needs to be validated in independent studies.

The top test-wise significant finding of this study was that OPC survivors with at least one allelic variant A in SNP rs6546481 or at least one variant allele G in SNP rs4854546 mapped to the *ANTXR1* gene had an increased risk of reporting moderate to severe xerostomia. *ANTXR1* is a protein coding gene possibly involved in cellular attachment and migration^38^. Furthermore, *ANTXR1* has also been associated with genome RNAi human phenotypes with a potential role in HPV16 infection^38^. Interestingly, this gene is also known to be associated with oral disorders such as oropharyngeal anthrax, which may result in oral mucosal infection, and gingival hypertrophy with gingival enlargement potentially due to inflammatory mechanisms, pharmaceutical treatment, and systemic conditions^38^. Therefore, *ANTXR1* may also have a critical role in risk of developing xerostomia. However, independent validation and subsequent functional studies are needed to elucidate role of the genetic variants identified in our study.

Our study also reported that *NLRP9* was potentially associated with increased risk of reporting moderate to severe xerostomia. Prior studies have linked *NLRP9* to urothelial carcinoma^46^. More importantly the *NLRP9* gene encodes a protein that can potentially regulate the innate immune system and may play a vital role in inflammatory response^46^. *NLRP9*, along with other genes including *PYCARD* and *CASP1*, is involved in the formation of inflammasome for activation with subsequent cytokine release to trigger or mediate the inflammatory response^42^. It is important to note that inflammation is postulated to contribute to acute xerostomia during and immediately after HNC treatment^5,6^, and our findings of a potential association between *NLRP9* and xerostomia may provide some evidence to support this hypothesis.

*RTP1* was associated with increased risk of reporting moderate to severe xerostomia. It is a protein coding gene involved in expression of bitter taste receptors in the circumvallate papillae of the tongue^47^. This gene plays a role in binding of olfactory receptors^44^ which may play a role in tumorigenesis with development and progression of melanoma^48^, invasive breast cancer^49^, and endometrial cancer^50^. Olfactory receptors may also be a potential biomarker for patient outcomes and tumor cell proliferation in prostate cancer^48^.

*GLT1D1* was also associated with higher risk of reporting moderate to severe xerostomia. *GLT1D1* is a protein coding gene involved in transferase activity including transfer of glycosyl groups^43^. GenomeRNAi phenotypes for this gene include HPV16 pseudovirus infection^51^. Liu et al. recently reported that *GLT1D1* was involved in programmed cell death and was identified as a biomarker predictive of poor prognosis among patients with B-cell non-Hodgkin lymphoma^52^.

*EGFLAM*, a protein coding gene which plays a role in matrix assembly and cell adhesiveness was associated with higher risk of reporting moderate to severe xerostomia. It is considered a biomarker in some cancers including a hypomethylated tumor maker in ovarian cancer^53^ and with altered expression in non-Hodgkin lymphoma^54^. Importantly, expression of this gene correlates with cell proliferation, migration, invasion, and poor prognosis by activating the P13/AKT pathway in Glioblastoma^55^.

Another gene, *SHROOM3*, was suggestively associated with an increased risk of moderate to severe xerostomia. *SHROOM3* is a protein coding gene that may play a role in the regulation of cell shape changes in some tissues and binding of actin, proteins, and beta-catenins and actin filament and ligand-gated sodium channel activity^41^. This gene is known to be associated with several disorders including acute myeloid leukemia^41^. Furthermore, a previous study reported that *SHROOM3* was associated with chronic kidney disease with high levels of oxidatively damaged DNA and genomic instability^56^. Finally, several additional SNPs were suggestive of risk lowering or risk enhancing for xerostomia, although, they did not map to any gene or function except one which was linked with a ncRNA class RNA gene.

Limitations of our study include the small sample size and associated low power; thus, the study must be considered as preliminary and exploratory. Consequently, none of the genetic variants reached the genome-wide significance level of 5.0 × 10^–8^. Larger genomic studies and subsequent functional studies are needed to independently validate our findings. Furthermore, information on baseline xerostomia was not available in our study and data on mean parotid gland dose was not available for all patients.

In conclusion, our novel albeit preliminary exploratory study identified 15 genetic variants suggestively associated with moderate to severe xerostomia among OPC survivors, suggesting multifactorial genetic etiology of the dry mouth symptoms. Studies to further elucidate the role of genetic susceptibility to xerostomia can inform the development of risk stratification and clinical interventions for targeted prevention, surveillance and management to alleviate the devastating impact of xerostomia and improve quality of life in OPC survivors.

## Supplementary Information

Supplementary Information.

## Data Availability

Genomewide genotyping data have been deposited in dbGaP (Study Accession number phs001173.v1.p1).
