# Supplementary material for: Genetic susceptibility to patient-reported xerostomia among long-term oropharyngeal cancer survivors
Source: Sci Rep. 2022 Apr 22;12:6662. doi: 10.1038/s41598-022-10538-9 (PMC9033773; doi:10.1038/s41598-022-10538-9)
Supplement: Supplementary file 1 — Supplementary Information. [file 41598_2022_10538_MOESM1_ESM.docx]

**Supplementary Table 1:** Top 100 SNPs Associated with Moderate to Severe Xerostomia Among Oropharyngeal Cancer Survivors in Our Study (*P* < 1 × 10^-4^).

| **SNP** | **Chromosome Position** | **Base-Pair Position** | **Minor Allele** | **OR** | **L95** | **U95** | ***P*** | **Frequency of Minor Allele in Moderate to Severe Xerostomia** | **Frequency of Minor Allele in None to Mild Xerostomia** | **Gene** |
| --- | --- | --- | --- | --- | --- | --- | --- | --- | --- | --- |
| rs6546481 | 2 | 69,313,511 | A | 4.70 | 2.50 | 8.83 | 4.3x10-^7^ | 0.21 | 0.05 | *ANTXR1* (anthrax toxin receptor) |
| rs4854546 | 2 | 69,317,925 | G | 4.70 | 2.50 | 8.83 | 4.3x10^-7^ | 0.21 | 0.05 | *ANTXR1* (anthrax toxin receptor) |
| rs16903936 | 5 | 38,322,975 | G | 3.98 | 2.16 | 7.31 | 5.1x10^-6^ | 0.20 | 0.06 | *EGFLAM (EGF Like, Fibronectin Type III and Laminin G Domains):* intron variant |
| rs10518156 | 4 | 77,695,104 | G | 6.65 | 2.65 | 16.69 | 7.1x10^-6^ | 0.13 | 0.02 | *SHROOM3* (shroom family member 3) |
| rs746154 | 15 | 70,677,754 | A | 0.31 | 0.18 | 0.52 | 8.0x10^-6^ | 0.31 | 0.59 | NA |
| rs1038553 | 15 | 53,679,121 | G | 0.27 | 0.14 | 0.50 | 9.5x10^-6^ | 0.07 | 0.22 | NA |
| rs4776140 | 15 | 53,680,596 | G | 0.27 | 0.15 | 0.51 | 1.5x10^-5^ | 0.07 | 0.22 | *LOC105370826:* 2KB upstream variant |
| rs11882068 | 19 | 56,227,165 | G | 3.53 | 1.96 | 6.37 | 1.7x10^-5^ | 0.20 | 0.07 | *NLRP9 (*NLR family pyrin domain containing 9) |
| rs4760542 | 12 | 129,385,824 | G | 2.94 | 1.80 | 4.80 | 1.8x10^-05^ | 0.27 | 0.11 | *GLT1D1* (glycosyltransferase 1 domain containing 1) |
| rs3014269 | 1 | 227,539,205 | G | 4.08 | 2.11 | 7.89 | 2.1x10^-5^ | 0.18 | 0.05 | NA |
| rs7523492 | 1 | 157,637,964 | G | 0.42 | 0.28 | 0.64 | 2.5x10^-5^ | 0.26 | 0.46 | NA |
| rs1486548 | 15 | 53,665,247 | A | 0.30 | 0.17 | 0.54 | 2.7x10^-5^ | 0.08 | 0.23 | NA |
| rs11714564 | 3 | 186,918,213 | G | 3.05 | 1.80 | 5.17 | 2.9x10^-5^ | 0.24 | 0.09 | *RTP1*: Receptor Transporter Protein 1,3 Prime UTR Variant *LOC101929106*: Intron Variant |
| rs10219117 | 10 | 92,022,480 | A | 0.39 | 0.24 | 0.61 | 3.1x10^-5^ | 0.17 | 0.35 | NA |
| rs12565883 | 1 | 20,883,203 | G | 0.38 | 0.24 | 0.61 | 3.3x10^-5^ | 0.15 | 0.33 | NA |
| rs1158267 | 11 | 44,396,537 | A | 5.06 | 2.30 | 11.12 | 3.5x10^-5^ | 0.24 | 0.06 | NA |
| rs17339367 | 5 | 38,338,761 | A | 3.93 | 2.03 | 7.62 | 3.7x10^-5^ | 0.17 | 0.05 | *EGFLAM*: Intron Variant |
| rs359955 | 1 | 33,134,774 | A | 0.35 | 0.21 | 0.59 | 4.0x10^-5^ | 0.12 | 0.27 | *RBBP4*: Intron Variant |
| rs8031474 | 15 | 70,683,274 | G | 0.34 | 0.20 | 0.57 | 4.5x10^-5^ | 0.35 | 0.61 | NA |
| rs6860569 | 5 | 169,781,990 | A | 2.30 | 1.55 | 3.41 | 5.0x10^-5^ | 0.45 | 0.26 | *KCNIP1*: Intron Variant |
| rs10076027 | 5 | 172,956,807 | G | 0.42 | 0.28 | 0.64 | 5.0x10^-5^ | 0.22 | 0.40 | NA |
| rs10916077 | 1 | 227,212,024 | A | 3.65 | 1.91 | 6.99 | 5.4x10^-5^ | 0.17 | 0.05 | *CDC42BPA*: Intron Variant |
| rs16835131 | 1 | 33,148,935 | A | 0.22 | 0.10 | 0.51 | 5.5x10^-5^ | 0.04 | 0.15 | *SYNC*: Intron Variant,  RBBP4: 3 Prime UTR Variant |
| rs17727145 | 19 | 44,874,369 | A | 2.92 | 1.73 | 4.93 | 5.6x10^-5^ | 0.24 | 0.10 | NA |
| rs13152864 | 5 | 68,003,493 | A | 0.45 | 0.31 | 0.66 | 5.7x10^-5^ | 0.34 | 0.53 | NA |
| rs3852710 | 16 | 82,603,752 | A | 6.41 | 2.35 | 17.50 | 6.0x10^-5^ | 0.10 | 0.02 | *LOC101928392*: Intron Variant |
| rs5011374 | 20 | 9,212,186 | A | 2.17 | 1.48 | 3.17 | 6.3x10^-5^ | 0.59 | 0.40 | *PLCB4:* intron variant |
| rs2206716 | 11 | 34,443,816 | A | 2.40 | 1.56 | 3.69 | 6.8x10^-5^ | 0.35 | 0.18 | NA |
| rs2765501 | 1 | 157,804,648 | A | 0.45 | 0.30 | 0.66 | 6.8x10^-5^ | 0.29 | 0.48 | *CD5L*: Intron Variant |
| rs1453391 | 11 | 34,585,751 | A | 0.30 | 0.16 | 0.56 | 6.9x10^-5^ | 0.17 | 0.41 | NA |
| rs6494863 | 15 | 70,671,899 | A | 0.35 | 0.21 | 0.59 | 7.4x10^-5^ | 0.35 | 0.60 | NA |
| rs10169997 | 2 | 74,194,587 | A | 3.48 | 1.89 | 6.41 | 7.6x10^-5^ | 0.35 | 0.13 | *DGUOK-AS1*: Intron Variant |
| rs3843010 | 3 | 186,937,047 | A | 2.19 | 1.49 | 3.22 | 8.5 x10^-5^ | 0.49 | 0.31 | *MASP1*: 3 Prime UTR Variant |
| rs2241349 | 5 | 179,260,009 | A | 0.32 | 0.17 | 0.57 | 8.6x10^-5^ | 0.19 | 0.43 | *SQSTM1*: Intron Variant |
| rs1437909 | 2 | 133,797,169 | G | 2.13 | 1.46 | 3.12 | 8.8x10^-5^ | 0.55 | 0.37 | *NCKAP5*: Intron Variant |
| rs306487 | 19 | 56,483,192 | G | 2.21 | 1.49 | 3.27 | 9.0x10^-5^ | 0.46 | 0.28 | *NLRP8*: Intron Variant |
| rs11741878 | 5 | 159,961,053 | A | 0.21 | 0.09 | 0.50 | 9.3x10^-5^ | 0.03 | 0.14 | NA |
| rs10514546 | 16 | 82,620,805 | A | 5.12 | 2.13 | 12.32 | 9.3x10^-5^ | 0.12 | 0.02 | NA |
| rs9578030 | 13 | 31,399,506 | A | 0.40 | 0.25 | 0.64 | 9.6x10^-5^ | 0.16 | 0.33 | NA |
| rs4435491 | 2 | 69,328,883 | A | 2.61 | 1.61 | 4.25 | 1.0x10^-4^ | 0.26 | 0.12 | *ANTXR1*: Intron Variant *MIR3126*: 2KB Upstream Variant |
| rs2954870 | 8 | 75,832,460 | G | 0.42 | 0.27 | 0.65 | 1.1x10^-4^ | 0.19 | 0.36 | NA |
| rs6869108 | 5 | 103,042,676 | A | 0.35 | 0.20 | 0.59 | 1.1x10^-4^ | 0.29 | 0.54 | *NALOC105379107:* intron variant |
| rs6789637 | 3 | 170,201,640 | A | 2.15 | 1.46 | 3.17 | 1.2x10^-4^ | 0.47 | 0.29 | *SLC7A14*: Intron Variant *SLC7A14-AS1*: Intron Variant |
| rs11735775 | 4 | 77,649,374 | G | 3.25 | 1.77 | 5.97 | 1.2x10^-4^ | 0.18 | 0.06 | *SHROOM3:* intron variant |
| rs2366981 | 5 | 38,378,483 | A | 4.39 | 1.98 | 9.72 | 1.3x10^-4^ | 0.13 | 0.03 | *EGFLAM*: Intron Variant |
| rs1871163 | 2 | 38,441,983 | G | 2.17 | 1.46 | 3.21 | 1.3x10^-4^ | 0.44 | *0.27* | *LOC102723739:* Intron Variant |
| rs4769189 | 13 | 22,647,376 | G | 0.38 | 0.23 | 0.63 | 1.3x10^-4^ | 0.13 | 0.28 | *LOC105370108*: Intron Variant |
| rs4771432 | 13 | 103,151,179 | A | 0.25 | 0.11 | 0.54 | 1.3x10^-4^ | 0.04 | 0.16 | NA |
| rs1861939 | 3 | 170,198,967 | G | 2.12 | 1.45 | 3.12 | 1.4x10^-4^ | 0.49 | 0.32 | *SLC7A14*: Intron Variant *SLC7A14-AS1*: Intron Variant |
| rs8105479 | 19 | 44,668,377 | G | 0.32 | 0.18 | 0.59 | 1.4x10^-4^ | 0.18 | 0.41 | *ZNF226*: 2KB Upstream Variant |
| rs7413116 | 1 | 182,057,506 | A | 0.46 | 0.31 | 0.69 | 1.4x10^-4^ | 0.27 | 0.45 | NA |
| rs7627412 | 3 | 147,502,818 | A | 2.40 | 1.54 | 3.76 | 1.4x10^-4^ | 0.31 | 0.16 | NA |
| rs11064838 | 12 | 120,021,728 | G | 2.09 | 1.43 | 3.05 | 1.4x10^-4^ | 0.62 | 0.44 | *LOC105370027:* intron variant |
| rs5752454 | 22 | 27,361,417 | A | 3.13 | 1.74 | 5.62 | 1.5x10^-4^ | 0.37 | 0.16 | NA |
| rs10183412 | 2 | 782,581 | A | 2.17 | 1.46 | 3.24 | 1.5x10^-4^ | 0.42 | 0.25 | *LINC01115:* intron variant |
| rs4674113 | 2 | 217,638,521 | G | 3.33 | 1.79 | 6.19 | 1.5x10^-4^ | 0.18 | 0.06 | *LOC101928278*: Intron Variant |
| rs10118855 | 9 | 126,889,267 | A | 0.35 | 0.20 | 0.61 | 1.6x10^-4^ | 0.26 | 0.49 | NA |
| rs6691569 | 1 | 157,648,098 | A | 2.23 | 1.48 | 3.35 | 1.6x10^-4^ | 0.40 | *0.23* | *FCRL3:* Intron Variant |
| rs9508459 | 13 | 30,024,572 | A | 0.47 | 0.32 | 0.70 | 1.6x10^-4^ | 0.30 | 0.48 | *MTUS2* |
| rs7078039 | 10 | 56,622,131 | G | 0.38 | 0.22 | 0.63 | 1.6x10^-4^ | 0.12 | 0.27 | *PCDH15*: Intron Variant |
| rs7169159 | 15 | 53,671,945 | A | 0.21 | 0.08 | 0.50 | 1.7x10^-4^ | 0.06 | 0.24 | NA |
| rs1534480 | 2 | 28,838,527 | A | 3.05 | 1.69 | 5.49 | 1.7x10^-4^ | 0.19 | 0.07 | *PLB1:* intron variant |
| rs698092 | 3 | 186,969,634 | G | 2.10 | 1.43 | 3.09 | 1.8x10^-4^ | 0.48 | 0.30 | *MASP1*: Intron Variant |
| rs11037969 | 11 | 44,389,241 | G | 4.76 | 2.08 | 10.88 | 1.8x10^-4^ | 0.21 | 0.05 | NA |
| rs4142656 | 2 | 38,443,883 | G | 2.19 | 1.46 | 3.27 | 1.9x10^-4^ | 0.43 | 0.25 | *LOC102723739*: Intron Variant |
| rs696562 | 9 | 109,809,366 | A | 0.47 | 0.31 | 0.70 | 1.9x10^-4^ | 0.27 | 0.45 | *LOC340512*: Intron Variant |
| rs4857401 | 3 | 98,271,961 | G | 0.34 | 0.19 | 0.61 | 2.0x10^-4^ | 0.09 | 0.22 | NA |
| rs1045893 | 1 | 201,939,752 | A | 2.72 | 1.61 | 4.58 | 2.0x10^-4^ | 0.51 | 0.28 | *TIMM17A:* 3 prime UTR variant |
| rs2511477 | 11 | 67,918,678 | A | 4.32 | 2.00 | 9.33 | 2.0x10^-4^ | 0.23 | 0.07 | NA |
| rs9581094 | 13 | 25,082,630 | G | 3.46 | 1.80 | 6.65 | 2.0x10^-4^ | 0.30 | 0.11 | *PARP4*: Intron Variant *LOC105370117*: Intron Variant |
| rs1006444 | 2 | 133,787,580 | G | 2.08 | 1.41 | 3.05 | 2.0x10^-4^ | 0.49 | 0.32 | *NCKAP5:* Intron Variant |
| rs3121983 | 1 | 71,028,815 | A | 0.25 | 0.11 | 0.55 | 2.1x10^-4^ | 0.08 | 0.27 | NA |
| rs12285345 | 11 | 32,549,624 | G | 0.29 | 0.15 | 0.58 | 2.1x10^-4^ | 0.12 | 0.32 | NA |
| rs12412511 | 10 | 10,559,645 | C | 0.45 | 0.29 | 0.68 | 2.1x10^-4^ | 0.21 | 0.38 | *CELF2*: Intron Variant |
| rs6564080 | 16 | 84,854,245 | G | 2.97 | 1.68 | 5.25 | 2.2x10^-4^ | 0.40 | 0.18 | *CRISPLD2*: Intron Variant |
| rs7447161 | 5 | 107,899,940 | A | 3.39 | 1.79 | 6.44 | 2.2x10^-4^ | 0.43 | 0.18 | NA |
| rs9598200 | 13 | 61,500,362 | A | 0.37 | 0.21 | 0.63 | 2.3x10^-4^ | 0.10 | 0.24 | *LINC01442*: Intron Variant |
| rs12280779 | 11 | 95,251,008 | G | 2.84 | 1.64 | 4.92 | 2.3x10^-4^ | 0.21 | 0.09 | *LOC105369440*: Non-Coding Transcript Variant |
| rs331482 | 11 | 36,459,378 | A | 0.42 | 0.27 | 0.67 | 2.3x10^-4^ | 0.17 | 0.33 | *PRR5L*: Intron Variant |
| rs12662217 | 6 | 13,854,317 | G | 2.08 | 1.41 | 3.07 | 2.3x10^-4^ | 0.46 | 0.29 | NA |
| rs10497340 | 2 | 169,285,488 | G | 2.44 | 1.52 | 3.93 | 2.3x10^-4^ | 0.27 | 0.13 | *LOC102724081*: Non-Coding Transcript Variant |
| rs17472426 | 5 | 159,694,357 | C | 0.15 | 0.05 | 0.51 | 2.4x10^-4^ | 0.02 | 0.10 | *CCNJL*: Intron Variant *LOC105377691*: Intron Variant |
| rs3732042 | 2 | 68,519,877 | A | 11.40 | 2.52 | 51.67 | 2.4x10^-4^ | 0.17 | 0.02 | *CNRIP1:* Intron Variant *LOC107985892*: Intron Variant |
| rs2206138 | 20 | 9,252,390 | G | 2.10 | 1.43 | 3.10 | 2.4x10^-4^ | 0.47 | 0.29 | *PLCB4*: Intron Variant |
| rs12002347 | 9 | 18,513,871 | G | 0.23 | 0.09 | 0.55 | 2.4x10^-4^ | 0.03 | 0.13 | *ADAMTSL1*: Intron Variant |
| rs1769461 | 14 | 87,504,911 | A | 0.47 | 0.31 | 0.71 | 2.5x10^-4^ | 0.26 | 0.43 | NA |
| rs9861194 | 3 | 40,309,225 | A | 3.52 | 1.81 | 6.86 | 2.5x10^-4^ | 0.39 | 0.16 | *EIF1B-AS1*: Intron Variant |
| rs17306659 | 2 | 156,589,930 | A | 0.29 | 0.14 | 0.59 | 2.5x10^-4^ | 0.05 | 0.17 | NA |
| rs1955277 | 5 | 63,079,104 | A | 2.06 | 1.41 | 3.01 | 2.5x10^-4^ | 0.54 | 0.36 | NA |
| rs365344 | 19 | 27,862,694 | A | 2.62 | 1.56 | 4.40 | 2.5x10^-4^ | 0.53 | 0.30 | NA |
| rs399246 | 19 | 30,595,364 | A | 2.03 | 1.39 | 2.97 | 2.6x10^-4^ | 0.53 | 0.36 | NA |
| rs1253393 | 10 | 99,009,969 | C | 0.18 | 0.06 | 0.51 | 2.6x10^-4^ | 0.02 | 0.11 | *ARHGAP19*: Intron Variant *ARHGAP19-SLIT1*: Intron Variant |
| rs184435 | 5 | 104,073,794 | G | 4.47 | 1.93 | 10.32 | 2.6x10^-4^ | 0.12 | 0.03 | *LOC105379109*: Intron Variant |
| rs2407365 | 19 | 27,893,661 | C | 2.75 | 1.62 | 4.66 | 2.7x10^-4^ | 0.52 | 0.28 | NA |
| rs7251096 | 19 | 35,848,955 | A | 2.35 | 1.49 | 3.69 | 2.7x10^-4^ | 0.30 | 0.15 | *FFAR3*: 2KB Upstream Variant |
| rs11176338 | 12 | 67,023,408 | A | 0.40 | 0.24 | 0.66 | 2.8x10^-4^ | 0.13 | 0.27 | *GRIP1*: Intron Variant |
| rs12412502 | 10 | 129,761,287 | A | 0.31 | 0.16 | 0.60 | 2.8x10^-4^ | 0.07 | 0.18 | *PTPRE*: Intron Variant |
| rs9579645 | 13 | 31,310,506 | C | 0.31 | 0.16 | 0.60 | 2.8x10^-4^ | 0.07 | 0.18 | *ALOX5AP:* intron variant |
| rs4881483 | 10 | 5,610,349 | G | 0.46 | 0.31 | 0.70 | 2.9x10^-4^ | 0.25 | 0.42 | *LOC105376381*: 2KB Upstream Variant |
| rs6557634 | 8 | 23,060,256 | G | 0.49 | 0.34 | 0.72 | 2.9x10^-4^ | 0.35 | 0.52 | *TNFRSF10A*: Missense Variant |

**Supplementary Table 2:** Results for top 25 gene set using the pathway-driven GSEA-SNP Analysis

| **Gene set name** | **Size** | **Normalized enrichment**  **score** | **Nominal p-val** | **FDR p-val** | **FWER p-val** |
| --- | --- | --- | --- | --- | --- |
| ACEVEDO_METHYLATED_IN_LIVER_CANCER_DN | 173 | 1.13 | 0.19 | 1.00 | 0.78 |
| MEISSNER_BRAIN_HCP_WITH_H3K4ME3_AND_H3K27ME3 | 173 | 1.13 | 0.19 | 1.00 | 0.78 |
| GOBP_TISSUE_DEVELOPMENT | 173 | 1.13 | 0.19 | 1.00 | 0.78 |
| chr5p13 | 76 | 1.12 | 0.24 | 1.00 | 0.79 |
| BLANCO_MELO_BRONCHIAL_EPITHELIAL_CELLS_INFLUENZA_A_DEL_NS1_INFECTION_UP | 76 | 1.12 | 0.24 | 1.00 | 0.79 |
| PEREZ_TP53_TARGETS | 76 | 1.12 | 0.24 | 1.00 | 0.79 |
| KIM_MYC_AMPLIFICATION_TARGETS_UP | 76 | 1.12 | 0.24 | 1.00 | 0.79 |
| BENPORATH_SUZ12_TARGETS | 76 | 1.12 | 0.24 | 1.00 | 0.79 |
| BENPORATH_ES_WITH_H3K27ME3 | 76 | 1.12 | 0.24 | 1.00 | 0.79 |
| MEISSNER_NPC_HCP_WITH_H3K4ME2_AND_H3K27ME3 | 76 | 1.12 | 0.24 | 1.00 | 0.79 |
| MIKKELSEN_NPC_HCP_WITH_H3K4ME3_AND_H3K27ME3 | 76 | 1.12 | 0.24 | 1.00 | 0.79 |
| NABA_ECM_GLYCOPROTEINS | 76 | 1.12 | 0.24 | 1.00 | 0.79 |
| NABA_CORE_MATRISOME | 76 | 1.12 | 0.24 | 1.00 | 0.79 |
| NABA_MATRISOME | 76 | 1.12 | 0.24 | 1.00 | 0.79 |
| LEE_BMP2_TARGETS_UP | 76 | 1.12 | 0.24 | 1.00 | 0.79 |
| LIU_SMARCA4_TARGETS | 76 | 1.12 | 0.24 | 1.00 | 0.79 |
| MIR12123 | 76 | 1.12 | 0.24 | 1.00 | 0.79 |
| MIR1283 | 76 | 1.12 | 0.24 | 1.00 | 0.79 |
| MIR7110_3P | 76 | 1.12 | 0.24 | 1.00 | 0.79 |
| MIR4755_5P | 76 | 1.12 | 0.24 | 1.00 | 0.79 |
| MIR5006_3P | 76 | 1.12 | 0.24 | 1.00 | 0.79 |
| MIR8063 | 76 | 1.12 | 0.24 | 1.00 | 0.79 |
| MIR4692 | 76 | 1.12 | 0.24 | 1.00 | 0.79 |
| MIR20A_3P | 76 | 1.12 | 0.24 | 1.00 | 0.79 |
| MIR6071 | 76 | 1.12 | 0.24 | 1.00 | 0.79 |

**Supplementary Table 3:** Top 50 SNPs Identified From Association Analysis of Mild to Severe Versus None Xerostomia Among Oropharyngeal Cancer Survivors in Our Study.

| **SNP** | **Chromosome Position** | **Base-Pair Position** | **Minor Allele** | **OR** | **L95** | **U95** | ***P*** | **Frequency of Minor Allele in Moderate to Severe Xerostomia** | **Frequency of Minor Allele in None to Mild Xerostomia** |
| --- | --- | --- | --- | --- | --- | --- | --- | --- | --- |
| rs899860 | 6 | 130,086,475 | A | 0.19 | 0.10 | 0.35 | 2.84x10^-7^ | 0.17 | 0.52 |
| rs11163293 | 1 | 81,897,255 | G | 0.11 | 0.05 | 0.25 | 3.02x10^-7^ | 0.10 | 0.50 |
| rs7111291 | 11 | 6,666,267 | A | 5.69 | 2.50 | 12.98 | 2.41x10^-6^ | 0.49 | 0.15 |
| rs2802865 | 1 | 55,719,127 | A | 0.21 | 0.11 | 0.40 | 6.27x10^-6^ | 0.13 | 0.42 |
| rs3852052 | 3 | 186,924,035 | G | 0.14 | 0.05 | 0.38 | 8.59x10^-6^ | 0.43 | 0.84 |
| rs131737 | 22 | 51,040,546 | G | 0.25 | 0.13 | 0.47 | 9.42x10^-6^ | 0.31 | 0.65 |
| rs4917864 | 10 | 101,149,298 | A | 0.20 | 0.10 | 0.39 | 1.37x10^-5^ | 0.09 | 0.33 |
| rs9355143 | 6 | 168,289,199 | A | 6.60 | 2.33 | 18.72 | 1.88x10^-5^ | 0.38 | 0.08 |
| rs10945454 | 6 | 168,304,320 | A | 6.60 | 2.33 | 18.72 | 1.88x10^-5^ | 0.38 | 0.08 |
| rs762672 | 22 | 51,064,818 | A | 0.25 | 0.13 | 0.46 | 1.92x10^-5^ | 0.19 | 0.48 |
| rs131724 | 22 | 51,055,900 | G | 0.25 | 0.14 | 0.47 | 2.18x10^-5^ | 0.19 | 0.48 |
| rs9728976 | 1 | 82,130,153 | G | 0.19 | 0.09 | 0.40 | 2.38x10^-5^ | 0.19 | 0.56 |
| rs29465 | 7 | 111,630,024 | A | 0.27 | 0.14 | 0.50 | 2.90x10^-5^ | 0.35 | 0.67 |
| rs4754136 | 11 | 105,717,038 | A | 0.20 | 0.10 | 0.41 | 3.08x10^-5^ | 0.08 | 0.31 |
| rs219649 | 21 | 27,824,285 | A | 6.53 | 2.43 | 17.55 | 4.12x10^-5^ | 0.55 | 0.16 |
| rs3773982 | 3 | 190,280,052 | A | 0.25 | 0.14 | 0.48 | 4.46x10^-5^ | 0.15 | 0.42 |
| rs131718 | 22 | 51,057,923 | G | 0.27 | 0.14 | 0.52 | 4.61x10^-5^ | 0.40 | 0.71 |
| rs2408239 | 21 | 23,332,626 | A | 0.19 | 0.09 | 0.39 | 4.70x10^-5^ | 0.06 | 0.27 |
| rs2177843 | 10 | 75,409,877 | A | 0.22 | 0.11 | 0.44 | 5.07x10^-5^ | 0.10 | 0.33 |
| rs7308615 | 12 | 114,981,387 | A | 0.20 | 0.09 | 0.43 | 5.88x10^-5^ | 0.16 | 0.50 |
| rs16887197 | 8 | 116,246,667 | A | 0.20 | 0.09 | 0.41 | 6.67x10^-5^ | 0.07 | 0.27 |
| rs2094071 | 10 | 100,037,683 | G | 0.20 | 0.09 | 0.41 | 6.67x10^-5^ | 0.07 | 0.27 |
| rs4447263 | 12 | 125,434,580 | G | 0.28 | 0.15 | 0.53 | 7.75x10^-5^ | 0.38 | 0.69 |
| rs4765004 | 12 | 125,437,974 | A | 0.28 | 0.15 | 0.54 | 8.15x10^-5^ | 0.38 | 0.69 |
| rs4516060 | 12 | 125,438,516 | A | 0.28 | 0.15 | 0.54 | 8.15x10^-5^ | 0.38 | 0.69 |
| rs2286250 | 2 | 159,518,352 | A | 0.22 | 0.11 | 0.44 | 8.91x10^-5^ | 0.08 | 0.29 |
| rs650465 | 1 | 193,832,544 | A | 0.19 | 0.09 | 0.42 | 9.31x10^-5^ | 0.13 | 0.44 |
| rs1029589 | 7 | 42,055,725 | A | 0.19 | 0.09 | 0.42 | 9.31x10^-5^ | 0.13 | 0.44 |
| rs11766565 | 7 | 16,312,057 | G | 0.28 | 0.14 | 0.54 | 9.57x10^-5^ | 0.43 | 0.73 |
| rs1120073 | 11 | 28,977,527 | G | 0.14 | 0.06 | 0.36 | 1.03x10^-4^ | 0.06 | 0.31 |
| rs4234091 | 2 | 241,911,027 | A | 0.27 | 0.14 | 0.50 | 1.05x10^-4^ | 0.16 | 0.42 |
| rs2301533 | 6 | 168,323,664 | G | 5.82 | 2.05 | 16.53 | 1.15x10^-4^ | 0.35 | 0.08 |
| rs11822168 | 11 | 105,630,803 | A | 0.22 | 0.11 | 0.45 | 1.15x10^-4^ | 0.08 | 0.29 |
| rs2226567 | 11 | 105,681,713 | G | 0.22 | 0.11 | 0.45 | 1.15x10^-4^ | 0.08 | 0.29 |
| rs1998006 | 20 | 1,374,364 | G | 0.18 | 0.08 | 0.40 | 1.16x10^-4^ | 0.05 | 0.23 |
| rs11621897 | 14 | 61,705,553 | G | 9.34 | 2.18 | 40.02 | 1.25x10^-4^ | 0.38 | 0.06 |
| rs1877089 | 4 | 24,195,219 | G | 0.25 | 0.13 | 0.49 | 1.35x10^-4^ | 0.11 | 0.33 |
| rs6506598 | 18 | 8,563,622 | G | 0.29 | 0.16 | 0.55 | 1.38x10^-4^ | 0.37 | 0.67 |
| rs3606 | 17 | 70,909,455 | A | 0.30 | 0.16 | 0.56 | 1.39x10^-4^ | 0.30 | 0.58 |
| rs2278420 | 19 | 52,471,872 | G | 0.26 | 0.14 | 0.51 | 1.47x10^-4^ | 0.13 | 0.35 |
| rs959167 | 15 | 93,978,414 | G | 0.09 | 0.03 | 0.28 | 1.51x10^-4^ | 0.07 | 0.44 |
| rs38903 | 7 | 116,890,886 | A | 8.37 | 2.00 | 35.06 | 1.52x10^-4^ | 0.27 | 0.04 |
| rs1754511 | 1 | 57,438,919 | A | 0.28 | 0.15 | 0.53 | 1.57x10^-4^ | 0.17 | 0.42 |
| rs853807 | 5 | 67,752,638 | A | 7.32 | 2.17 | 24.72 | 1.57x10^-4^ | 0.43 | 0.09 |
| rs12324454 | 15 | 35,121,225 | A | 0.15 | 0.06 | 0.38 | 1.58x10^-4^ | 0.06 | 0.31 |
| rs7834295 | 8 | 11,001,301 | C | 0.25 | 0.13 | 0.50 | 1.59x10^-4^ | 0.11 | 0.33 |
| rs9890738 | 17 | 35,029,700 | A | 0.19 | 0.08 | 0.42 | 1.61x10^-4^ | 0.05 | 0.23 |
| rs7341280 | 6 | 150,860,123 | A | 0.31 | 0.17 | 0.57 | 1.65x10^-4^ | 0.30 | 0.58 |
| rs12698887 | 7 | 69,739,690 | A | 0.21 | 0.09 | 0.45 | 1.66x10^-4^ | 0.14 | 0.44 |
| rs6478890 | 9 | 132,085,237 | A | 0.30 | 0.16 | 0.57 | 1.67x10^-4^ | 0.40 | 0.69 |

**Supplementary Table 4:** Top 50 SNPs Identified From Association Analysis of Moderate to Severe Versus None Xerostomia Among Oropharyngeal Cancer Survivors in Our Study.

| **SNP** | **Chromosome Position** | **Base-Pair Position** | **Minor Allele** | **OR** | **L95** | **U95** | ***P*** | **Frequency of Minor Allele in Moderate to Severe Xerostomia** | **Frequency of Minor Allele in None to Mild Xerostomia** |
| --- | --- | --- | --- | --- | --- | --- | --- | --- | --- |
| rs11163293 | 1 | 81,897,255 | G | 0.05 | 0.02 | 0.17 | 4.90x10^-8^ | 0.05 | 0.50 |
| rs7111291 | 11 | 6,666,267 | A | 6.26 | 2.67 | 14.68 | 2.75x10^-6^ | 0.52 | 0.15 |
| rs2802865 | 1 | 55,719,127 | A | 0.17 | 0.08 | 0.36 | 4.49x10^-6^ | 0.11 | 0.42 |
| rs4447263 | 12 | 125,434,580 | G | 0.21 | 0.11 | 0.42 | 5.18x10^-6^ | 0.32 | 0.69 |
| rs4765004 | 12 | 125,437,974 | A | 0.21 | 0.11 | 0.42 | 5.18x10^-6^ | 0.32 | 0.69 |
| rs4516060 | 12 | 125,438,516 | A | 0.21 | 0.11 | 0.42 | 5.18x10^-6^ | 0.32 | 0.69 |
| rs16887197 | 8 | 116,246,667 | A | 0.11 | 0.04 | 0.29 | 8.28x10^-6^ | 0.04 | 0.27 |
| rs4234091 | 2 | 241,911,027 | A | 0.19 | 0.09 | 0.40 | 1.24x10^-5^ | 0.12 | 0.42 |
| rs4754136 | 11 | 105,717,038 | A | 0.16 | 0.07 | 0.36 | 2.21x10^-5^ | 0.07 | 0.31 |
| rs13068877 | 3 | 2,657,255 | A | 0.16 | 0.06 | 0.40 | 3.36x10^-5^ | 0.36 | 0.78 |
| rs899860 | 6 | 130,086,475 | A | 0.23 | 0.12 | 0.46 | 3.44x10^-5^ | 0.20 | 0.52 |
| rs3852052 | 3 | 186,924,035 | G | 0.14 | 0.05 | 0.39 | 3.56x10^-5^ | 0.43 | 0.84 |
| rs131737 | 22 | 51,040,546 | G | 0.25 | 0.13 | 0.49 | 4.50x10^-5^ | 0.31 | 0.65 |
| rs6506598 | 18 | 8,563,622 | G | 0.25 | 0.13 | 0.49 | 5.39x10^-5^ | 0.34 | 0.67 |
| rs3773982 | 3 | 190,280,052 | A | 0.22 | 0.11 | 0.45 | 5.57x10^-5^ | 0.14 | 0.42 |
| rs746154 | 15 | 70,677,754 | A | 0.17 | 0.07 | 0.42 | 5.89x10^-5^ | 0.31 | 0.72 |
| rs2177843 | 10 | 75,409,877 | A | 0.19 | 0.09 | 0.42 | 6.45x10^-5^ | 0.09 | 0.33 |
| rs4758443 | 11 | 6,648,424 | A | 5.23 | 2.11 | 12.95 | 7.06x10^-5^ | 0.44 | 0.13 |
| rs17410294 | 1 | 55,538,462 | A | 0.18 | 0.08 | 0.42 | 7.12x10^-5^ | 0.08 | 0.31 |
| rs615652 | 1 | 55,567,410 | A | 0.18 | 0.08 | 0.42 | 7.12x10^-5^ | 0.08 | 0.31 |
| rs1165222 | 1 | 55,638,075 | G | 0.18 | 0.08 | 0.42 | 7.12x10^-5^ | 0.08 | 0.31 |
| rs11031821 | 11 | 32,517,406 | A | 0.15 | 0.06 | 0.38 | 7.16x10^-5^ | 0.12 | 0.47 |
| rs219649 | 21 | 27,824,285 | A | 6.91 | 2.46 | 19.43 | 7.18x10^-5^ | 0.56 | 0.16 |
| rs7791525 | 7 | 128,732,013 | A | 0.16 | 0.06 | 0.41 | 7.20x10^-5^ | 0.37 | 0.78 |
| rs2094071 | 10 | 100,037,683 | G | 0.16 | 0.06 | 0.39 | 7.41x10^-5^ | 0.05 | 0.27 |
| rs2408239 | 21 | 23,332,626 | A | 0.16 | 0.06 | 0.39 | 7.41x10^-5^ | 0.05 | 0.27 |
| rs9273363 | 6 | 32,626,272 | A | 0.18 | 0.07 | 0.42 | 7.71x10^-5^ | 0.18 | 0.56 |
| rs12698887 | 7 | 69,739,690 | A | 0.15 | 0.06 | 0.38 | 8.35x10^-5^ | 0.10 | 0.44 |
| rs12494352 | 3 | 2,655,046 | G | 0.17 | 0.07 | 0.43 | 8.41x10^-5^ | 0.38 | 0.78 |
| rs2703257 | 8 | 5,773,243 | G | 0.27 | 0.14 | 0.52 | 8.82x10^-5^ | 0.29 | 0.60 |
| rs12272563 | 11 | 32,509,232 | A | 0.16 | 0.06 | 0.40 | 8.93x10^-5^ | 0.12 | 0.47 |
| rs161641 | 5 | 104,096,630 | G | 7.56 | 2.26 | 25.32 | 9.67x10^-5^ | 0.34 | 0.06 |
| rs10050461 | 5 | 104,118,033 | G | 7.56 | 2.26 | 25.32 | 9.67x10^-5^ | 0.34 | 0.06 |
| rs853807 | 5 | 67,752,638 | A | 8.55 | 2.44 | 29.94 | 1.07x10^-4^ | 0.47 | 0.09 |
| rs131724 | 22 | 51,055,900 | G | 0.25 | 0.13 | 0.49 | 1.10x10^-4^ | 0.19 | 0.48 |
| rs762672 | 22 | 51,064,818 | A | 0.25 | 0.13 | 0.49 | 1.10x10^-4^ | 0.19 | 0.48 |
| rs9410340 | 9 | 91,399,821 | C | 0.27 | 0.14 | 0.52 | 1.10x10^-4^ | 0.26 | 0.56 |
| rs9375383 | 6 | 125,354,154 | C | 0.26 | 0.14 | 0.52 | 1.16x10^-4^ | 0.35 | 0.67 |
| rs1120073 | 11 | 28,977,527 | G | 0.09 | 0.03 | 0.33 | 1.16x10^-4^ | 0.04 | 0.31 |
| rs9890738 | 17 | 35,029,700 | A | 0.13 | 0.05 | 0.37 | 1.17x10^-4^ | 0.04 | 0.23 |
| rs2659871 | 11 | 6,652,618 | A | 4.38 | 1.94 | 9.88 | 1.23x10^-4^ | 0.47 | 0.17 |
| rs8065610 | 17 | 15,175,570 | A | 0.27 | 0.14 | 0.52 | 1.23x10^-4^ | 0.27 | 0.58 |
| rs4710656 | 6 | 68,221,990 | G | 0.19 | 0.08 | 0.45 | 1.34x10^-4^ | 0.30 | 0.69 |
| rs2277814 | 21 | 47,409,503 | A | 0.17 | 0.07 | 0.42 | 1.37x10^-4^ | 0.06 | 0.27 |
| rs892523 | 7 | 1,583,288 | A | 0.28 | 0.14 | 0.54 | 1.43x10^-4^ | 0.36 | 0.67 |
| rs974644 | 7 | 8,396,670 | A | 0.15 | 0.06 | 0.40 | 1.45x10^-4^ | 0.09 | 0.41 |
| rs9355143 | 6 | 168,289,199 | A | 6.11 | 2.10 | 17.77 | 1.46x10^-4^ | 0.36 | 0.08 |
| rs10945454 | 6 | 168,304,320 | A | 6.11 | 2.10 | 17.77 | 1.46x10^-4^ | 0.36 | 0.08 |
| rs12482177 | 21 | 47,403,435 | G | 0.23 | 0.11 | 0.48 | 1.48x10^-4^ | 0.12 | 0.38 |
| rs7851181 | 9 | 91,392,349 | G | 0.24 | 0.12 | 0.50 | 1.51x10^-4^ | 0.14 | 0.40 |

**Supplementary Table 5:** Results of top 15 SNPs Associated with Moderate to Severe Dysphagia Among Oropharyngeal Cancer Survivors in Our Study.

| **SNP** | **Chromosome Position** | **Base-Pair Position** | **Minor Allele** | **OR** | **L95** | **U95** | ***P*** | **Frequency of Minor Allele in Moderate to Severe Xerostomia** | **Frequency of Minor Allele in None to Mild Xerostomia** |
| --- | --- | --- | --- | --- | --- | --- | --- | --- | --- |
| rs1158267 | 11 | 44,396,537 | A | 3.31 | 1.70 | 6.46 | 9.4x10^-4^ | 0.25 | 0.09 |
| rs746154 | 15 | 70,677,754 | A | 0.52 | 0.31 | 0.90 | 2.4x10^-2^ | 0.35 | 0.50 |
| rs7523492 | 1 | 157,637,964 | G | 0.64 | 0.44 | 0.95 | 3.0x10^-2^ | 0.31 | 0.41 |
| rs4760542 | 12 | 129,385,824 | G | 1.63 | 1.05 | 2.53 | 4.1x10^-2^ | 0.24 | 0.16 |
| rs6546481 | 2 | 69,313,511 | A | 1.64 | 0.98 | 2.75 | 6.4x10^-2^ | 0.16 | 0.10 |
| rs3014269 | 1 | 227,539,205 | G | 1.41 | 0.79 | 2.49 | 2.8x10^-1^ | 0.12 | 0.09 |
| rs11882068 | 19 | 56,227,165 | G | 1.28 | 0.75 | 2.20 | 3.9x10^-1^ | 0.13 | 0.11 |
| rs10219117 | 10 | 92,022,480 | A | 0.82 | 0.54 | 1.24 | 4.1x10^-1^ | 0.24 | 0.28 |
| rs10518156 | 4 | 77,695,104 | G | 1.25 | 0.64 | 2.45 | 4.8x10^-1^ | 0.08 | 0.07 |
| rs16903936 | 5 | 38,322,975 | G | 1.21 | 0.70 | 2.06 | 4.8x10^-1^ | 0.13 | 0.11 |
| rs11714564 | 3 | 186,918,213 | G | 1.16 | 0.72 | 1.88 | 5.3x10^-1^ | 0.17 | 0.15 |
| rs7461547 | 8 | 131,888,133 | A | 1.12 | 0.63 | 1.99 | 6.6x10^-1^ | 0.12 | 0.10 |
| rs1486548 | 15 | 53,665,247 | A | 0.90 | 0.56 | 1.46 | 7.2x10^-1^ | 0.17 | 0.18 |
| rs12565883 | 1 | 20,883,203 | G | 1.07 | 0.71 | 1.61 | 7.5x10^-1^ | 0.27 | 0.26 |
| rs4776140 | 15 | 53,680,596 | G | 0.92 | 0.57 | 1.50 | 8.1x10^-1^ | 0.16 | 0.17 |
